# Differential roles for ACBD4 and ACBD5 in peroxisome–ER interactions and lipid metabolism

**DOI:** 10.1016/j.jbc.2023.105013

**Published:** 2023-07-04

**Authors:** Joseph L. Costello, Janet Koster, Beatriz S.C. Silva, Harley L. Worthy, Tina A. Schrader, Christian Hacker, Josiah Passmore, Frans A. Kuypers, Hans R. Waterham, Michael Schrader

**Affiliations:** 1 Department of Biosciences, University of Exeter, Exeter, UK; 2 Department of Clinical Chemistry, Laboratory Genetic Metabolic Diseases, Amsterdam University Medical Center Location University of Amsterdam, Amsterdam, The Netherlands; 3 Luxembourg Centre for Systems Biomedicine, Campus Belval | House of Biomedicine II, Université du Luxembourg, Belvaux, Luxembourg; 4 Division of Cell Biology, Utrecht University, Utrecht, The Netherlands; 5 University of California, San Francisco, USA

**Keywords:** peroxisomes, ER, ACBD5, ACBD4, VAPB, membrane contact sites, fatty acid metabolism

## Abstract

Peroxisomes and the endoplasmic reticulum (ER) are intimately linked subcellular organelles, physically connected at membrane contact sites. While collaborating in lipid metabolism, for example, of very long-chain fatty acids (VLCFAs) and plasmalogens, the ER also plays a role in peroxisome biogenesis. Recent work identified tethering complexes on the ER and peroxisome membranes that connect the organelles. These include membrane contacts formed *via* interactions between the ER protein VAPB (vesicle-associated membrane protein-associated protein B) and the peroxisomal proteins ACBD4 and ACBD5 (acyl-coenzyme A-binding domain protein). Loss of ACBD5 has been shown to cause a significant reduction in peroxisome–ER contacts and accumulation of VLCFAs. However, the role of ACBD4 and the relative contribution these two proteins make to contact site formation and recruitment of VLCFAs to peroxisomes remain unclear. Here, we address these questions using a combination of molecular cell biology, biochemical, and lipidomics analyses following loss of ACBD4 or ACBD5 in HEK293 cells. We show that the tethering function of ACBD5 is not absolutely required for efficient peroxisomal β-oxidation of VLCFAs. We demonstrate that loss of ACBD4 does not reduce peroxisome–ER connections or result in the accumulation of VLCFAs. Instead, the loss of ACBD4 resulted in an increase in the rate of β-oxidation of VLCFAs. Finally, we observe an interaction between ACBD5 and ACBD4, independent of VAPB binding. Overall, our findings suggest that ACBD5 may act as a primary tether and VLCFA recruitment factor, whereas ACBD4 may have regulatory functions in peroxisomal lipid metabolism at the peroxisome–ER interface.

Acyl-CoA binding domain–containing proteins (ACBDs) are a large and diverse multigene family of proteins containing a conserved acyl-CoA binding motif (1, 2). ACBD proteins play key roles in controlling activated fatty acids, which are important lipid metabolites and regulate lipid metabolism and cellular signaling (3). In mammals, eight ACBD proteins (ACBD1-8) have been described, and recently, new functions for these proteins at organelle contact sites and as host interaction proteins for pathogens have been revealed (2).

ACBD4 and ACBD5 are C-terminally tail-anchored (TA) membrane proteins, which localize to peroxisomes and expose their N-terminal acyl-CoA binding domain to the cytosol (4). Peroxisomes are oxidative organelles with key roles in cellular reactive oxygen species and lipid metabolism, including fatty acid α- and β-oxidation and the synthesis of ether-phospholipids (*e.g.*, plasmalogens enriched in myelin sheaths). Many of these functions are performed in cooperation with other subcellular organelles such as mitochondria and the ER (5, 6). Defects in peroxisome biogenesis and metabolic function are linked to severe disorders with developmental and neurological defects (7, 8).

We, and others, previously showed that ACBD5 is involved in the tethering of peroxisomes to the ER as one component of a peroxisome-ER membrane contact site (9, 10). Peroxisomal ACBD5 interacts with ER-resident VAPB (vesicle-associated membrane protein (VAMP)–associated protein B) (11). The interaction is mediated by an FFAT-like motif (two phenylalanines (FF) in an acidic tract) in the central region of ACBD5, which binds to the major sperm protein (MSP) domain of VAPB. This interaction is regulated by phosphorylation of the ACBD5 FFAT-like motif (12). Co-expression of ACBD5 and VAPB increases peroxisome–ER interactions in mammalian cells while their depletion reduces contacts (9, 10). While causing a reduction of physical tethering between the ER and the peroxisomes, loss of ACBD5 also results in an increase in peroxisomal movement and a reduction in the expansion of the peroxisomal membrane, which is a requirement for the formation of peroxisomes by membrane growth and division (9, 13). Furthermore, increased levels of very long-chain fatty acids (VLCFAs) and alterations in plasmalogen and cholesterol levels were also observed in cells lacking ACBD5 (10, 14, 15). These findings indicate a role for the ACBD5-VAPB-mediated peroxisome–ER contacts in peroxisome biogenesis, membrane lipid transfer, and cooperative metabolism.

Several studies have now identified and characterized patients with mutations resulting in loss of ACBD5 (14, 15, 16, 17, 18, 19). ACBD5 deficiency results in progressive leukodystrophy, ataxia, progressive microcephaly with facial dysmorphisms, and retinal dystrophy. Lipid profiling of patient fibroblasts showed accumulation of VLCFAs, specifically C26:0, and an increased ratio of C26:0/C22:0, as well as a decrease in ether phospholipids, including plasmalogens (14). Several of these reports hypothesized a role for ACBD5 as a cofactor, potentially using its acyl-CoA binding capacity to facilitate the capture of peroxisomal VLCFA substrates for handover to the VLCFA transporter ABCD1, allowing import and subsequent β-oxidation in peroxisomes (15, 17). An ACBD5-deficient mouse model also displayed similarities with the ACBD5-deficient patient cell lines (20). The mice developed a progressive locomotor disorder with pathologic cerebellar alterations and showed increased VLCFAs and significantly altered peroxisome-ER associations.

Based on this work, we suggested that fatty acid synthesis and elongation at the ER and breakdown by peroxisomal β-oxidation are coordinated at the peroxisome–ER interface (21). This fits with the observation that the long-chain acyl-CoA synthetase ACSL1, which could potentially coordinate fatty-acid activation at such an interface, was identified as an interaction partner of ACBD5 and VAPB (22). However, the role that ACBD5 plays as a peroxisome–ER tethering factor and/or as a cofactor for VLCFA capture is unclear.

More recently, we identified ACBD4 as a second tail-anchored ACBD protein at peroxisomes and we showed that, like ACBD5, ACBD4 interacts with VAPB *via* an FFAT motif (12, 23). However, the specific roles of ACBD4 in peroxisome-ER interplay or lipid metabolism and if or how they differ from ACBD5 are presently unclear. A link between altered ACBD4 expression and both cardiac contraction and hepatocellular carcinoma has recently been reported (24, 25), although how this relates to the protein’s function is unknown.

To establish the roles of both ACBD5 and ACBD4 in peroxisome–ER interactions and lipid metabolism, we now combined molecular cell biology with biochemical and lipidomics analyses following the loss of ACBD4 or ACBD5. We observed that both ACBD4 and ACBD5 have the capacity to act as peroxisome–ER tethers and that expression of ACBD4 can compensate for reduced peroxisome–ER contacts when ACBD5 is lacking. However, we did not observe altered peroxisome–ER contacts in the absence of ACBD4. Lipid profiling of HEK293 cells that do not express ACBD4 or ACBD5 confirmed an increase in VLCFA-containing lipids in the absence of ACBD5. Complementation studies revealed that the tethering function of ACBD5 is not required for efficient peroxisomal β-oxidation of VLCFAs. Interestingly, and in contrast to the loss of ACBD5, the loss of ACBD4 resulted in an increase in the rate of β-oxidation of VLCFAs indicating that ACBD4 may have regulatory functions. The possible role of hetero- and homodimer formation of ACBD5 and ACBD4 in the regulation of peroxisomal lipid metabolism at the peroxisome–ER interface is discussed.

## Results

### ACBD4 resembles ACBD5 and can interact with VAPB to mediate peroxisome–ER contacts

**Figure 1:**
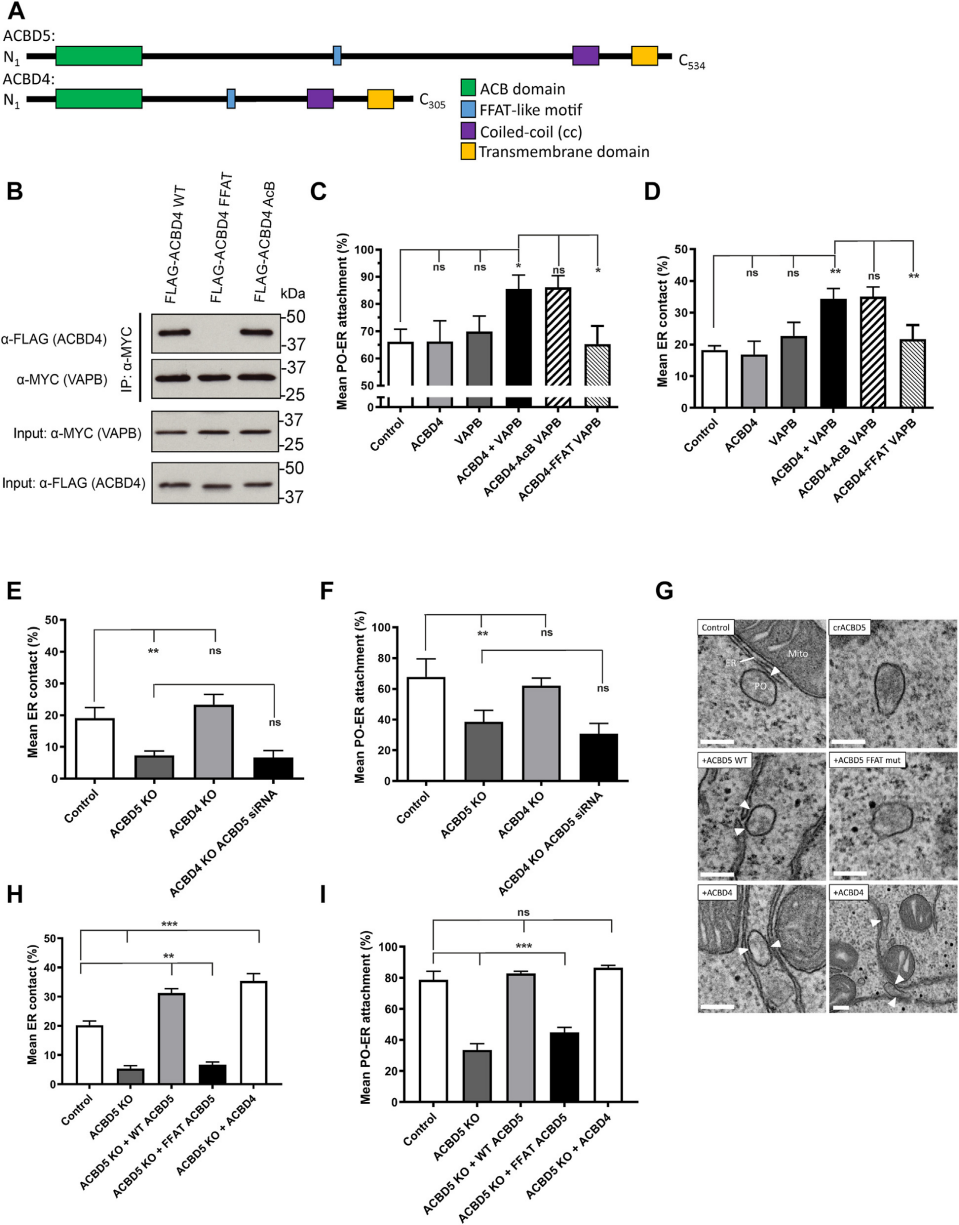
**ACBD4 resembles ACBD5 and can interact with VAPB to mediate peroxisome-ER contacts but loss of ACBD4 does not reduce contacts.***A*, protein architecture of ACBD5 and ACBD4 with known and predicted domains indicated. *B*, both FLAG-ACBD4 and Myc-VAPB were expressed in COS-7 cells and Myc-VAPB was immunoprecipitated and bound FLAG-ACBD4 detected by immunoblotting using FLAG/MYC antibodies (IP). Inputs represent 1% of total lysate. *C*, analysis of the mean population of peroxisomes associated with the ER (<15 nm) in COS-7 cells expressing the indicated proteins. *D*, assessment of the mean peroxisomal surface in contact with the ER in COS-7 cells expressing the indicated proteins. *E*, assessment of the mean peroxisomal surface in contact with the ER in control ACBD4 and ACBD5 KO HEK293 cells. *F*, analysis of the mean population of peroxisomes associated with the ER (<15 nm) in ACBD4/5 KO HEK293 cells. *G*, representative electron micrographs of peroxisome-ER interactions in the ACBD5 KO HEK293 FlpIn cells complemented with the indicted constructs. *H*, assessment of the mean peroxisomal surface in contact with the ER in ACBD5 KO HEK293 cells expressing the indicated proteins. *I*, analysis of the mean population of peroxisomes associated with the ER (<15 nm) in ACBD5 KO HEK293 cells expressing the indicated proteins. Data were analyzed by one-way ANOVA with Tukey’s multiple comparison test; ns, not significant; ∗∗*p* ≤ 0.01; ∗∗∗*p* ≤ 0.001. Error bars represent SD, with three to six experiments per condition and 56 ± 2 peroxisomes analyzed per experiment. Scale bars, (*G*) 200 nm. KO, CRISPR knock-out; Mito, mitochondrion; PO, peroxisomes.

**Supporting Figure S1:**
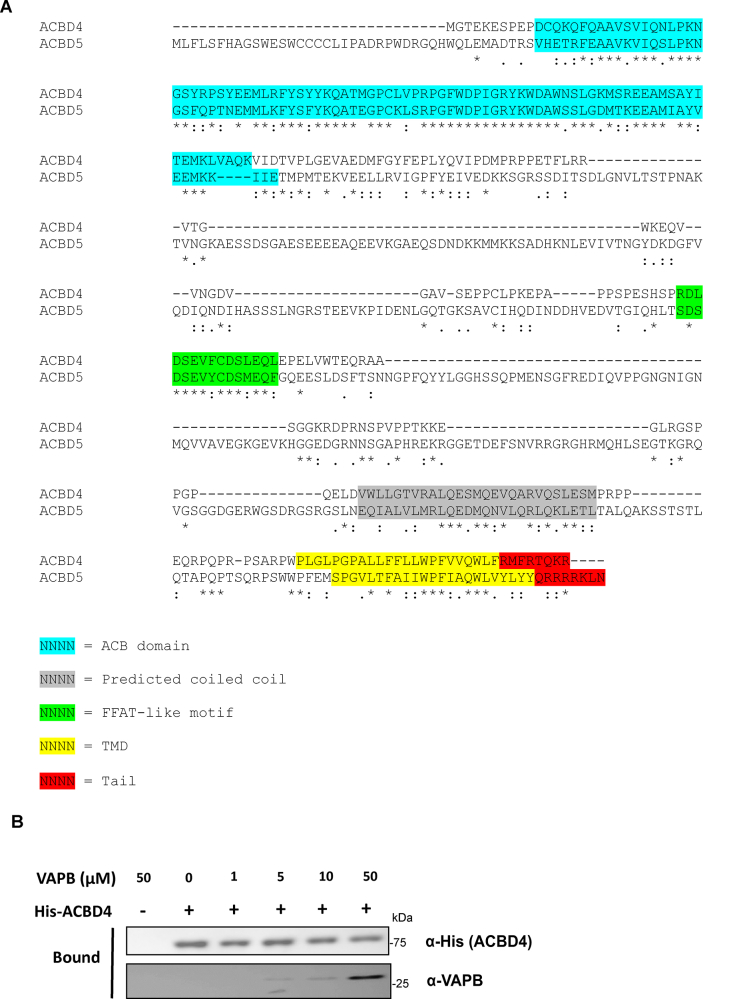
**Sequence alignment of human ACBD4 and ACBD5 and VAPB interaction of ACBD4 *in vitro*.***A*, alignment of human ACBD4 and ACBD5 with regions of interest highlighted. TMD: transmembrane domain, Tail: region following the TMD inside the organelle lumen. *B*, *in-vitro* binding of purified ACBD4 and VAPB. Recombinant versions of both proteins, lacking the TMD, were expressed in *E.coli* and purified. ACBD4 (10 μM) was bound onto Ni-NTA beads and incubated with indicated concentrations of VAPBmsp. Bound fractions were analysed by immunoblotting with His and VAPB antibodies.

Our previous work showed that overexpression of ACBD5 in combination with VAPB increased peroxisome–ER interactions in a manner dependent on the FFAT motif of ACBD5, while silencing or knock-out (KO) of ACBD5 reduced peroxisome-ER contacts (9, 26). ACBD4 and ACBD5 are structurally and phylogenetically closely related proteins with the same domain arrangement. They are both C-terminal tail-anchored proteins with an N-terminal ACB domain, a FFAT motif, and a predicted coiled-coil region (Fig. 1*A*). Overall, the proteins show 38.9% sequence similarity with the majority of similarity in the ACB domain (Fig. S1*A*). Based on sequence analysis, the FFAT motif of ACBD5 would be predicted to potentially allow stronger interaction with VAP than the FFAT motif of ACBD4 (27). However, unlike ACBD4, the interaction between ACBD5 and VAPB is dependent on the phosphorylation status of the ACBD5 FFAT motif (12).

The major apparent difference between the two proteins is that ACBD5 is a significantly larger protein than ACBD4 with a larger central region of unknown function. ACBD4 proteins are significantly more compact proteins, which appear to have evolved from ACBD5 by a later gene duplication, which only occurred in vertebrates (2).

To assess the role of ACBD4 in peroxisome–ER interactions, we first clarified that ACBD4 interacts with VAPB in a manner dependent on the FFAT motif of ACBD4, by mutating specific residues in the FFAT motif (12). We also determined that this interaction is not altered by specific mutation of key residues in the ACB domain, which disrupt lipid binding (28, 29). This was confirmed by immunoprecipitation (IP) following the co-expression of FLAG-ACBD4 and Myc-VAPB in COS-7 cells (Fig. 1*B*). We also confirmed that the binding between ACBD4 and VAPB was direct using recombinant proteins purified from *E. coli* (Fig. S1*B*).

To assess if the over-expression of ACBD4 and VAPB increased peroxisome-ER associations, we performed transmission electron microscopy (TEM) to quantify membrane contacts at the ultrastructural level. This is in line with our previous work using an unbiased spatial stereology approach as an effective way to measure changes in organelle interactions (9, 26). We determined both the average population of peroxisomes in close contact (<15 nm) with the ER (Fig. 1*C*: Mean attachment) and estimated the proportion of the peroxisomal surface closely opposed to the ER (Fig. 1*D*: Mean ER contact). Co-expression of wild-type or the ACB mutant of ACBD4 in combination with VAPB resulted in a significant increase in peroxisome ER contacts (Mean attachment: from ∼66% to 86%, Mean ER contact: from ∼19% to 35%). However, co-expression of an ACBD4 FFAT mutant in combination with VAPB resembled the expression of VAPB alone, with no significant increase in peroxisome–ER contacts observed. These findings support a role for ACBD4 in peroxisome–ER interactions, which is dependent on the FFAT motif of ACBD4 providing a binding site with VAPB to generate a tether. This increased tethering function for over-expressed ACBD4 is very similar to what was previously observed for ACBD5 (9).

### Loss of ACBD4 does not reduce peroxisome–ER contacts

Loss of a tethering protein often, but not always, results in a measurable loss of proximity between two organelles (30). In our previous studies, we observed a significant decrease in peroxisome–ER contacts when ACBD5 was silenced (for example a 24.5% decrease in Mean attachment in HEK293 cells) (9). To allow us to assess the impact of loss of ACBD4 on ER–peroxisome attachments, we generated HEK293 cell lines in which ACBD4 or ACBD5 were knocked-out and used EM to assess peroxisome-ER contact sites.

For the ACBD5 KO HEK293 cell line, we observed a significant decrease in both the mean attachment of peroxisomes to the ER and also the mean ER contact (reduced by ∼25% and 15% respectively) (Fig. 1, *E* and *F*). However, ACBD4 KO HEK293 cells showed no significant differences in peroxisome–ER contacts compared with controls. To test for a potential compensatory effect by ACBD5 under these conditions we also silenced ACBD5 in the ACBD4 KO HEK293 cell line. Here, the difference we observed in peroxisome-ER contacts was not significantly different from the loss of ACBD5 alone (Fig. 1, *E* and *F*). We conclude that in this cell line under our experimental conditions, loss of ACBD4 does not result in a decrease in peroxisome–ER associations. These findings indicate that ACBD5 is a major tether for peroxisome–ER interaction in HEK293 cells.

To assess if the over-expression of ACBD4 could complement the loss of ACBD5, we used the FlpIn system to generate HEK293 KO cell lines stably expressing ACBD4 or ACBD5. Here, as expected, overexpression of wild-type ACBD5 in an ACBD5 KO cell line restored peroxisome–ER contacts, and significantly increased the level of ER membrane contacts, whereas expression of an ACBD5-FFAT mutant did not (Fig. 1, G–I). Interestingly, overexpression of ACBD4 in the ACBD5 KO cells also restored peroxisome–ER contacts with a similar increase in ER membrane contacts (Fig. 1, G–I).

In combination, these results suggest that both ACBD4 and ACBD5 can mediate peroxisome-ER interactions by interacting with VAPB. However, only loss of ACBD5 and not loss of ACBD4 results in a decrease in peroxisome-ER tethering in HEK293 cells. This suggests that ACBD5 plays a more significant role in physically tethering the organelles under the conditions tested.

### VLCFA analyses following the loss of ACBD4 or ACBD5 reveal differential roles in lipid metabolism

**Figure 2:**
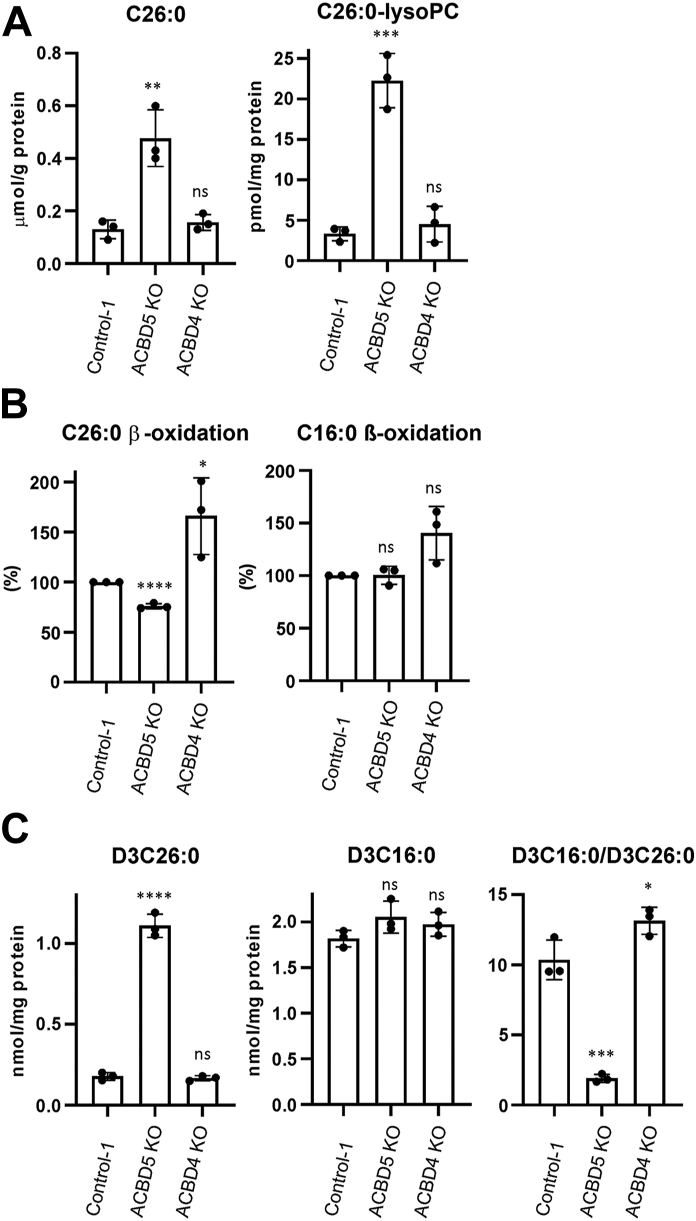
**VLCFA analysis in ACBD5 and ACBD4 KO HEK293 cells.***A*, analysis of levels of C26:0 and C26:0-lysoPC in control, ACBD4 and ACBD5 KO HEK293 cells. *B*, analysis of C26:0 and C16:0 β-oxidation (as a percentage relative to controls) in ACBD4, ACBD5 KO, and control HEK293 cells. *C*, D3-C22 loading assay with analysis of D3C26:0, D3C16:0, and the ratio of D3C16:0/D3C26:0 in control ACBD4 and ACBD5 KO HEK293 cells. Data analyzed by two-tailed unpaired *t* test; ns, not significant; ∗*p* ≤ 0.0332; ∗∗*p* ≤ 0.0021; ∗∗∗*p* ≤ 0.0002; ∗∗∗∗*p* ≤ 0.0001. Error bars represent SD, with three experiments per condition. KO, CRISPR knock-out.

To assess the contributions of ACBD5 and ACBD4 to lipid metabolism, we performed VLCFA analyses in control HEK293 and ACBD5 and ACBD4 KO HEK293 cell lines. Initially, we focused on assessing lipid species known to be altered by loss of ACBD5. In ACBD5 KO HEK293 cells, we observed an increase in C26:0 and C26:0-lysoPC levels, which are changes commonly observed in peroxisomal disorders including ACBD5 deficiency (Fig. 2*A*) (31). In line with this, a decrease in the beta-oxidation rate of C26:0 was also observed, while C16:0 beta-oxidation was unchanged (Fig. 2*B*). In a D3-C22:0 loading test (32) increased D3-C26:0 was detected, while D3-C16:0 levels remained the same, resulting in a significant decrease in the D3-C16:0/D3-C26:0 ratio (Fig. 2*C*). In this assay, cells are incubated with labeled D3-C22:0, which is exclusively beta-oxidized in peroxisomes, and assessment of the ratio of labeled metabolites can give an indication of rate of fatty acid elongation *versus* rate of peroxisomal beta-oxidation. A decreased D3-C16:0/D3-C26:0 ratio likely reflects increased chain elongation of the deuterium labeled C22:0 substrate *via* the ELOVL pathway on the ER, due to impairment of the peroxisomal beta-oxidation pathway (33). These results reflect previous observations in fibroblasts from a patient with ACBD5 deficiency (17). By comparison, in ACBD4 KO HEK293 cells there was no significant increase in VLCFA species C26:0 and C26:0-lyso PC, suggesting peroxisomal beta-oxidation was not reduced (Fig. 2*A*). Surprisingly, however, subsequent beta-oxidation rate analyses showed that loss of ACBD4 resulted in an increase in C26:0 beta-oxidation, with C16:0 beta-oxidation also apparently increased but not to a statistically significant extent (Fig. 2*B*). The D3-C22:0 loading test did not reveal significant changes in levels of labeled C26:0 and C16:0. However, the D3-C16:0/D3-C26:0 ratio was slightly increased—suggesting that the beta-oxidation of the D3-C22:0 substrate to C16:0 was increased in ACBD4 KO cells (Fig. 2*C*).

Overall, we observe differences in VLCFA processing following the loss of ACBD5 and ACBD4. ACBD5 KO HEK293 cells showed increased levels of VLCFAs and decreased beta-oxidation of C26:0, in line with previous studies on ACBD5 deficient cells. However, loss of ACBD4 resulted in unaltered levels of VLCFAs despite slightly increased rates of VLCFA beta-oxidation. To attempt to further explore these differences we next performed more detailed lipidomic analyses.

### Detailed lipidomic analyses of ACBD4 and ACBD5 KO HEK293 cells

**Figure 3:**
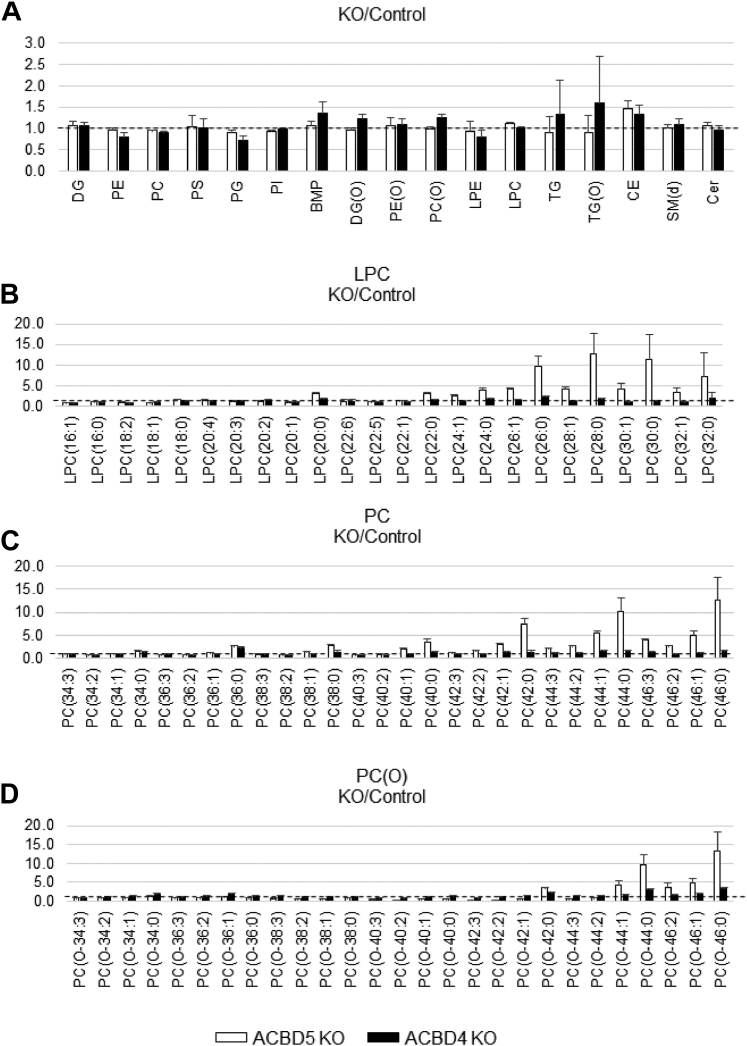
**Lipidomic analyses in ACBD5 and ACBD4 KO HEK293 cells.** Overview of major lipid species (*e.g.*, contains more than 40 lipid species) (*A*), and specific LPC (*B*), PC (*C*) and PC(O) (*D*) species of different chain lengths, plotted for ACBD5 or ACBD4 as ratio of control cells. For PC and PC(O) the lipid species with a chain length between 34 and 46 carbon atoms and with a maximum of three double bounds are displayed. The dotted line indicates the control value. KO, CRISPR knock-out.

Lipidomic profiling of the ACBD5 and ACBD4 KO HEK293 cells and comparing these with wild-type HEK293 cells revealed no major differences in the main lipid classes, including Phosphatidylcholine (PC), Phosphatidylethanolamine (PE), triglycerides (TG), Cholesterol esters (CE), lysoPC (LPC), ether (phospho)lipids, and sphingolipids (Fig 3*A*). However, closer inspection of individual lipid species in the ACBD5 KO HEK293 cells showed a relative increase in lipid species that contain saturated VLCFAs or, to a lesser extent, monounsaturated VLCFAs, including CE, LPC, PC, and PC (O) (Fig. 3, *B*–*D*), confirming earlier observations in ACBD5-deficient and peroxisome-deficient cells (14, 17).

In the ACBD4 KO cell lines, there were no significant differences in the major lipid classes as well as in the individual lipid species. Closer inspection of individual lipid species also did not show a similar pattern of significantly increased VLCFA-containing species as observed in the ACBD5 KO HEK293 cells (Fig. 3, *B*–*D*). Complete lipidomic analyses are shown in Supplementary File 1.

Overall, these findings indicate that the observed increase of individual lipid species containing saturated and monounsaturated VLCFAs in the ACBD5 KO HEK293 cells is due to the incorporation of VLCFAs, which accumulate due to the decreased peroxisomal beta-oxidation in these cells. This is similar to what is observed in peroxisome-deficient or peroxisomal beta-oxidation-deficient cells (14). The observed increased beta-oxidation in the ACBD4 KO cell line has no clear effect on the lipidome of HEK293 cells.

### The FFAT motif of ACBD5 is not required for efficient β-oxidation of VLCFAs

**Figure 4:**
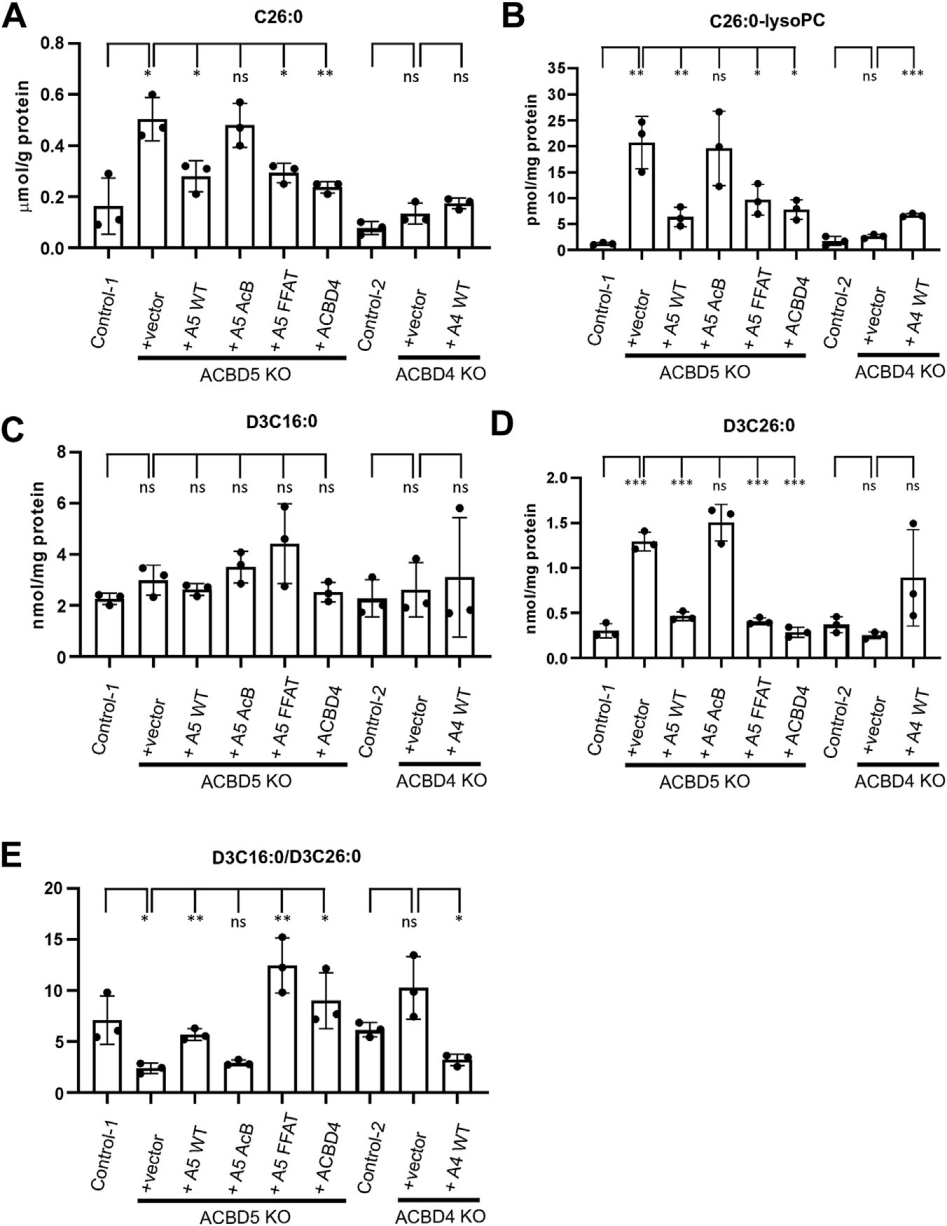
**VLCFA complementation analysis in ACBD5 and ACBD4 KO HEK293 cells.***A*, analysis of levels of C26:0 and (*B*) C26:0-lysoPC in control, ACBD4 and ACBD5 KO HEK293 cells complemented with the indicated proteins. D3-C22 loading assay with analysis of (*C*) D3C16:0, (*D*) D3C26:0, and (*E*) the ratio of D3C16:0/D3C26:0 in control, ACBD4 and ACBD5 KO HEK293 cells complemented with the indicated proteins. Data analysed by two-tailed unpaired *t* test; ns, not significant; ∗*p* ≤ 0.0332; ∗∗*p* ≤ 0.0021; ∗∗∗*p* ≤ 0.0002; ∗∗∗∗*p* ≤ 0.0001. Error bars represent SD, with three experiments per condition. Note: Control-2 cells contained the FlpIn site whilst Control-1 does not. A5, ACBD5; AcB, mutations in AcB domain; FFAT, mutations in FFAT motif; KO, CRISPR knock-out; WT, wild-type.

**Supporting Figure S2:**
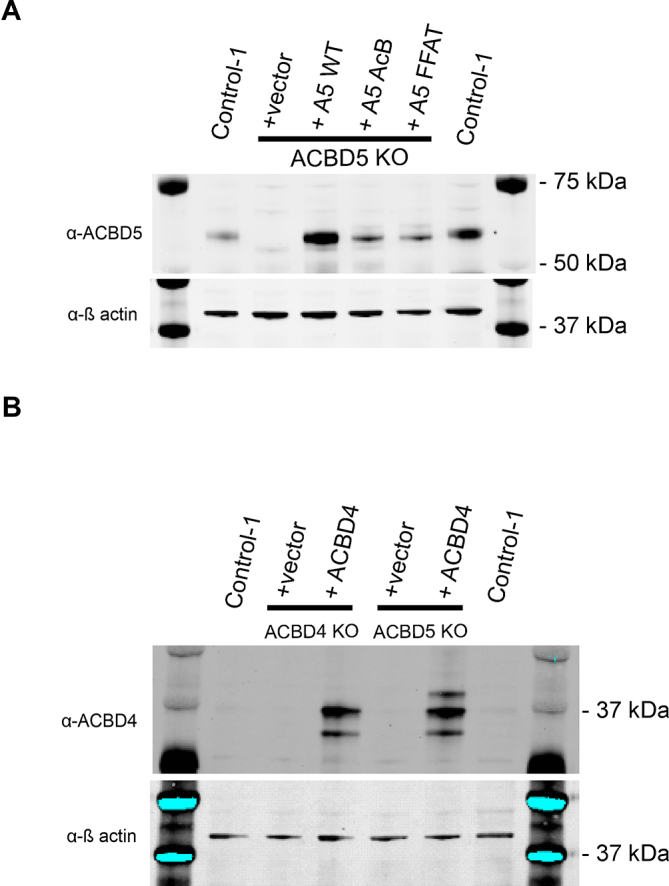
**Protein expression levels in HEK293 KO FlpIn cell lines.** Immunoblot analysis using indicated antibodies of (*A*) ACBD5 KO HEK293 cells complemented with wild-type ACBD5 and mutant ACBD5 proteins integrated with FlpIn system and (*B*) ACBD4 and ACBD5 KO HEK293 cells complemented with wild-type ACBD4 *via* FlpIn integration. β-actin was used as a loading control.

To test if ER tethering function or lipid binding capacity of ACBD5 resulted in the observed defects in VLCFA metabolism, we utilized the FlpIn system in our HEK293 KO cell lines to generate cell lines stably expressing wild-type ACBD5 or ACBD5 with mutations in either the ACB or FFAT motifs. Previously, we had shown that mutations in the FFAT motif disrupt ACBD4/5 binding to VAPB and reduce ER tethering capacity, whereas mutations in the ACB domain do not alter VAPB interaction (9) (Fig. 1). In ACBD5 KO HEK293 cells complemented with wild-type ACBD5 we observed significant complementation of lipid processing, with C26:0 and C26:0-lysoPC levels restored close to wild-type levels (Fig. 4, *A* and *B*). The same result was observed using the D3:C22:0 loading test, which showed that exogenous ACBD5 expression could almost fully complement the D3:C26:0 accumulation phenotype in the ACBD5 KO with D3:C26:0 levels and D3:C16:C26:0 ratio restored to close to normal control levels (Fig. 4, *C*–*E*). However, expression of ACBD5 with a mutated ACB domain did not result in complementation in any of these assays suggesting that the ACB domain is required for the proper metabolism of these VLCFAs. Interestingly, expression of the ACBD5 FFAT mutant, which is defective in ER tethering, did complement the defect and C26:0 was observed at near control levels. Similar results were observed when using the D3:C22:0 loading test. Although expression levels of the mutant forms of ACBD5 appeared less than the wild-type, the ACB mutant and FFAT mutant were expressed at similar levels (Fig. S2*A*). Overall, this suggests that ACBD5 with a functional ACB domain is required to prevent accumulation of C26:0 but tethering to the ER is not essential for this process to occur normally.

### ACBD4 expression can compensate for the loss of ACBD5 in VLCFA beta-oxidation but in the presence of ACBD5 has an inhibitory role

As ACBD4 could restore peroxisome-ER-tethering in ACBD5 KO cells we assessed if ACBD4 expression could also compensate for loss of ACBD5 in VLCFA beta-oxidation. Utilizing the HEK293 FlpIn system to express ACBD4 in ACBD5 KO cells, we observed that over-expression of wild-type ACBD4 in ACBD5 KO cells resulted in complementation of the D3-C22:0 processing defect and restored levels of C26:0 and C26:0-lyso-PC to the same extent as wild type ACBD5 (Fig. 4).

To assess the consequences of overexpression of ACBD4, in line with the hypothesis that ACBD4 may actually serve as a repressor of beta-oxidation (as its loss apparently increases beta-oxidation of VLCFAs) we also overexpressed ACBD4 in the ACBD4 KO HEK293 cells. As previously mentioned, knock-out of ACBD4 resulted in no significant alterations in C26:0 levels but did appear to increase beta-oxidation rates (Fig. 2). Following overexpression of ACBD4 we observed that whilst endogenous C26:0 levels were unaltered, there was a significant increase in the levels of C26:0-lysoPC compared with the controls (Fig. 4, *A* and *B*). There was also D3-C26:0 accumulation to a similar extent to that observed for KO of ACBD5 and there was a significantly altered ratio of D3-C16:0/D3-C26:0 compared to controls (Fig. 4, *C*–*E*). This is in contrast to ACBD4 or ACBD5 overexpression in ACBD5 KO cells, where we observed that overexpression of either protein reduced C26:0 accumulation in this assay, effectively complementing the KO phenotype (Fig. 4*A*). These results suggest that in the absence of ACBD5, ACBD4 expression may have a positive impact in restoring VCLFA metabolism defects. However, in the presence of ACBD5, increased levels of ACBD4 appear to induce defective VLCFA metabolism. This would be broadly in line with a role for ACBD4 as a repressor of ACBD5 VLCFA processing activity.

### ACBD4 and ACBD5 show different substrate specificity

**Figure 5:**
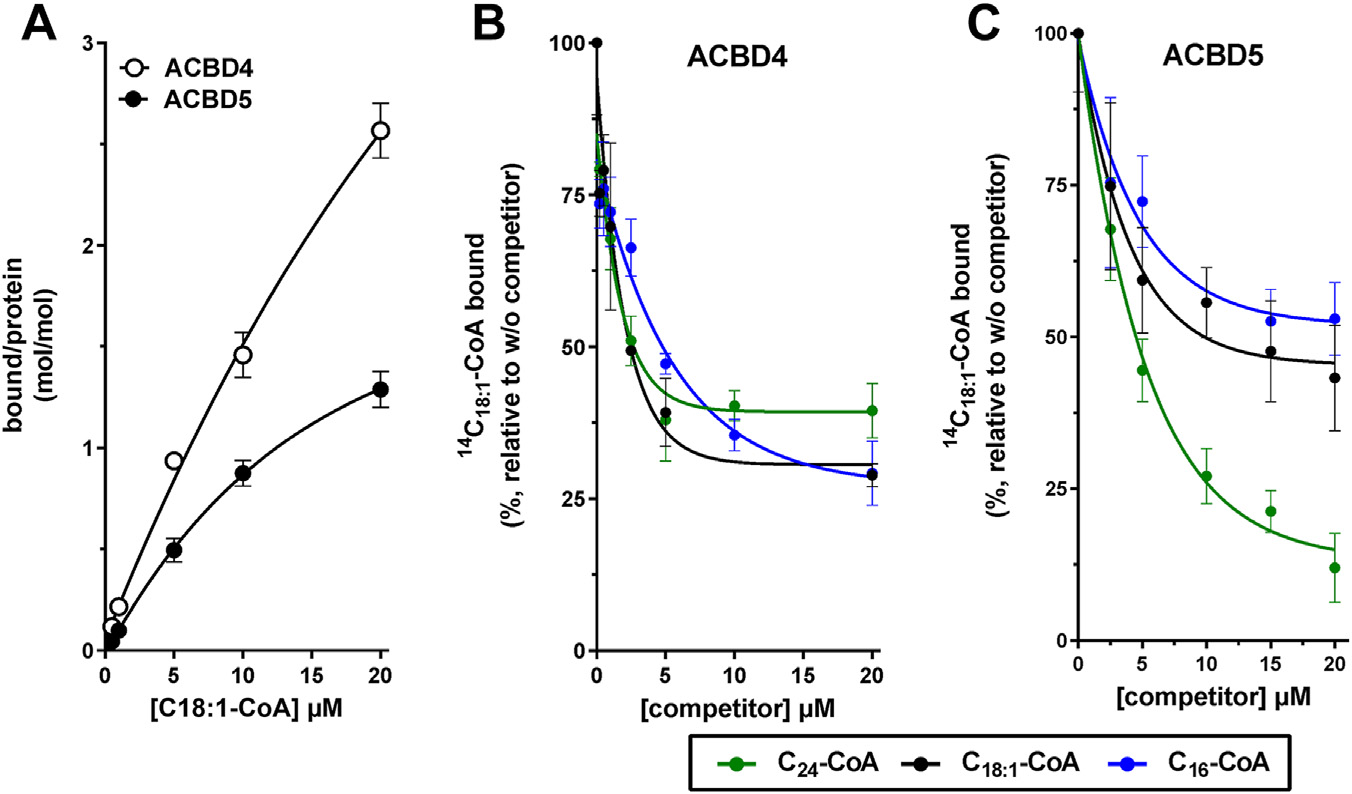
**Substrate preference of ACBD4 and ACBD5.***A*, binding activity was measured with 2 μM protein and increasing concentrations of 14C-C18:1-CoA (0.5–20 μM). Error bars represent the standard deviations of at least three measurements. Competition of the binding of 14C-C18:1-CoA (5 μM) was performed with increasing concentrations of C16-CoA, C18:1-CoA and C24-CoA (0.2–20 μM), as indicated to (*B*) ACBD4 and (*C*) ACBD5. Control reactions were performed in the absence of the competitors and values obtained in their presence are presented relative to the values obtained in their absence. Error bars in the four plots represent the standard deviations of three measurements.

As previously discussed, ACBD4 and ACBD5 are closely related proteins with similar, but not identical ACB domains (Fig. S1*A*). ACBD5 was previously suggested to have a preference for VLCFA-C26-CoA in an *in vitro* binding assay (15). However, no specific ligand binding data has been reported for ACBD4. To further assess possible differences between ACBD5 and ACBD4, recombinant forms of both proteins were produced in *E.coli* and acyl-CoA binding activity and acyl chain preference were assessed by binding competition of the radiolabelled [14C]C18:1-CoA by shorter and longer acyl-CoAs. The binding preference of a protein for an acyl-CoA can be measured by the efficiency of the displacement of the bound radiolabeled substrate with increasing concentrations of ligand competitor (34). For both ACBD4 and ACBD5, the addition of C16-CoA in the reaction resulted in the competition of [14C]C18:1-CoA binding to a level comparable with the addition of unlabeled C18:1-CoA, suggesting similar substrate preference for both ligands (Fig. 5, *A*–*C*). However, a stronger preference for the very-long-chain C24-CoA compared to the two long-chain acyl-CoAs (C18:1-CoA and C16-CoA) was observed for ACBD5 but not ACBD4, which displayed a similar preference for long and very-long acyl-CoAs.

This *in vitro* binding data confirmed the binding preference of ACBD5 for VLCFA-CoA, but also, indicates a significant difference in the properties of the ACB domains of ACBD4 and ACBD5.

### ACBD5 and ACBD4 form multimeric complexes

**Figure 6:**
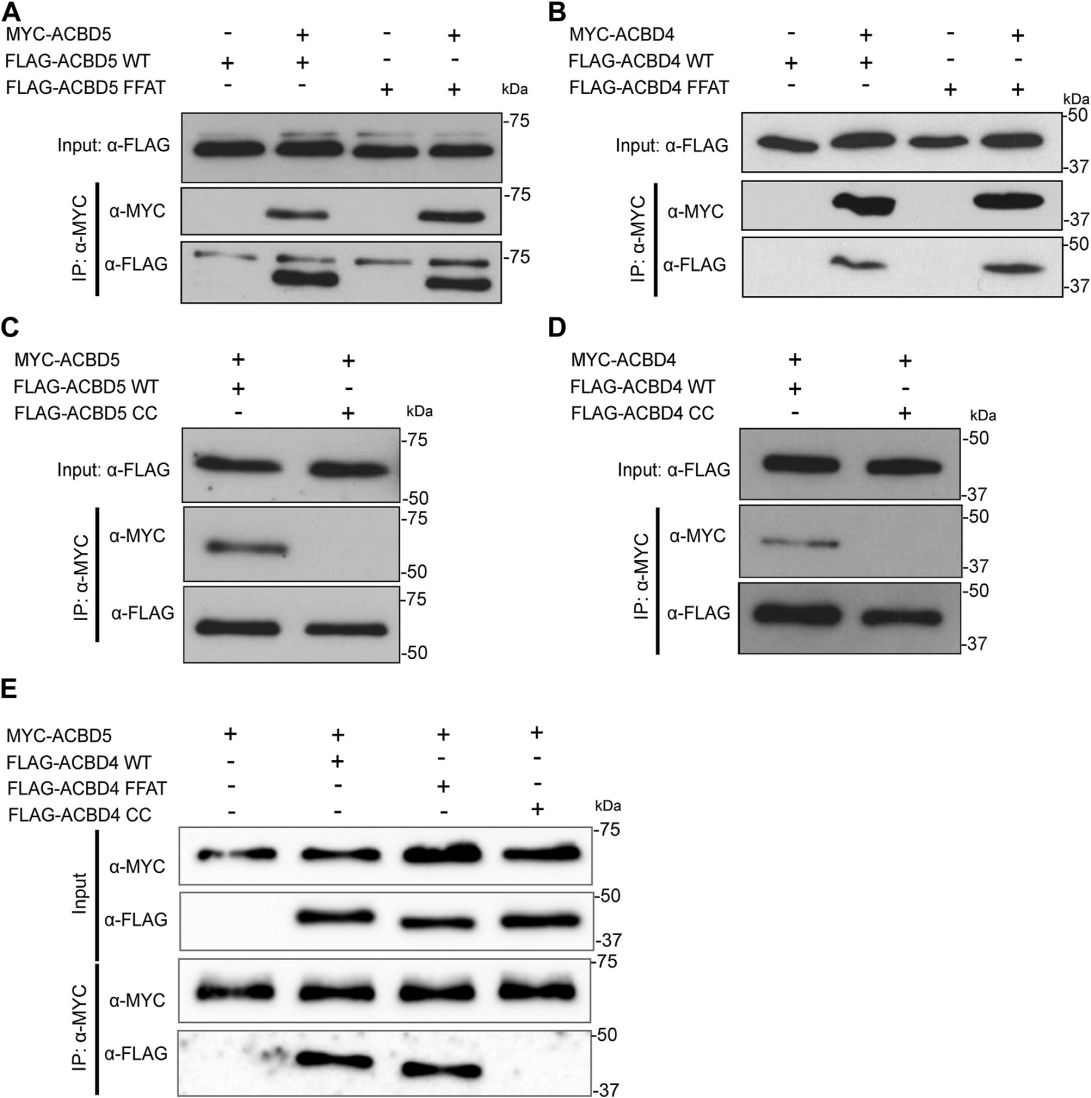
**Interactions between ACBD4 and ACBD5.***A*, FLAG-ACBD5, WT and FFAT mutants, and Myc-ACBD5, (*B*) FLAG-ACBD4, WT and FFAT mutants, and Myc-ACBD4, (*C*) FLAG-ACBD5, WT and CC mutants, and Myc-ACBD5, (*D*) FLAG-ACBD4, WT and CC mutants, and Myc-ACBD4 were expressed in COS-7 cells. Myc-ACBD4/5 was immunoprecipitated and bound FLAG-proteins detected by immunoblotting using FLAG/MYC antibodies. *E*, FLAG-ACBD4, WT, CC, and FFAT mutants, and Myc-ACBD5 were expressed in COS-7 cells. Myc-ACBD5 was immunoprecipitated and bound FLAG-proteins were detected by immunoblotting using FLAG/MYC antibodies. Inputs represent 1% of total lysate. CC, coiled-coil mutant; FFAT, FFAT mutant; IP, immunoprecipitation.

As our data suggested a potential inhibitory role for ACBD4 in the presence of ACBD5 we assessed the ability of both ACBD4 and ACBD5 to interact as potential homo- or heterodimers. Previously, dimer formation of ACBD5 has been suggested, but not demonstrated (15) and FFAT motif dimerization has also been associated with FFAT-VAP interaction but its significance remains unclear (35). As previously noted, ACBD4 and ACBD5 both contain a predicted coiled-coil domain (Fig. 1*A*), motifs that have frequently been observed to allow protein oligomerization (36). Therefore, we tested for potential coiled-coil mediated self-interaction of ACBD4/5, by co-expressing both FLAG and MYC-tagged versions of the proteins in COS-7 cells. We observed clear evidence of self-interaction for both ACBD5 and ACBD4 and in both cases, this was unchanged when the FFAT motif was mutated—suggesting the interaction was independent of binding to VAPB. (Fig. 6, *A* and *B*). To test if the coiled-coil motifs were involved in self-interaction, we mutated this region (ACBD4:M244P; ACBD5:M416P). We observed a significant reduction in self-interaction for both the ACBD4 and ACBD5 coiled-coil mutants compared with wild-type (Fig. 6, *C* and *D*). We conclude that both ACBD4 and ACBD5 can self-interact, dependent on the coiled-coil motif, and that this appears to be independent of the interaction with VAPB.

Finally, to test for the possibility of the formation of ACBD4-ACBD5 complexes we co-expressed both Myc-ACBD5 and FLAG-ACBD4 in COS-7 cells and tested for interaction between the two proteins. Myc-ACBD5 was able to precipitate both FLAG-ACBD4 wild-type and FLAG-ACBD4 with a mutated FFAT motif but not FLAG-ACBD4 with a mutated coiled coil. This suggests that ACBD5 and ACBD4 are able to interact with each other in a manner that depends on the coiled-coil region but is independent of the interaction with VAPB (Fig. 6*E*).

## Discussion

We show here that both ACBD5 and ACBD4 have the capacity to facilitate peroxisome-ER tethering as well as the potential to deliver VLCFAs to peroxisomes for beta-oxidation. However, whilst loss of ACBD5 causes a loss of peroxisome-ER interactions and defective processing of VLCFAs, loss of ACBD4 did not alter peroxisome-ER contacts and instead appeared to increase VLCFA beta-oxidation rates, albeit in a way that did not have a significant impact on the overall lipidome. We also demonstrated that the expression of ACBD4 can compensate for the loss of ACBD5 in both ER-peroxisome tethering and VLCFA processing. Finally, we have shown that the tethering capacity of ACBD5, its ability to bind VAPB and mediate peroxisome-ER association, was not required for its function in VLCFA processing.

By complementation analysis, we demonstrated that the tethering function of ACBD5 is not required to restore VLCFA processing in an ACBD5 KO HEK293 cell line. However, an intact ACB domain is required, as a mutant with defective lipid binding was unable to complement for loss of ACBD5 (Fig. 4). This suggests that the closer proximity to the ER provided by ACBD5-VAPB tethering is not necessary for VLCFA uptake by the peroxisomes and that the key role for ACBD5 in this process is likely to be as a lipid-binding cofactor for the VLCFA transporter ABCD1/ALDP on peroxisomes. These observations are reminiscent of a similar recent report on the mitochondrial-ER contact site protein PTPIP51 (37). PTPIP51 resembles ACBD5 in that it binds VAPB (and mediates ER-mitochondria tethering) and also contains a lipid-binding tetratricopeptide repeat (TPR) domain, which is able to bind phosphatidic acid (PA) (37). In their recent study, Yeo and colleagues demonstrated that mitochondrial cardiolipin, which can be generated at the inner mitochondrial membrane using PA supplied by the ER, is slightly reduced when PTPIP51 is depleted. Restoration of normal cardiolipin levels does not require the PTPIP51 FFAT motifs, suggesting that PTPIP51-mediated PA transfer from the ER to mitochondria does not require the ER-tethering function of PTPIP51. It is possible that the extent of contacts remaining following loss of tethers has an impact on these observations. Loss of ACBD5 only reduces and does not completely abolish ER–peroxisome interactions; in HEK293 ACBD5 KO cells ER–peroxisome contacts are only reduced by ∼50% (Fig. 1). While for PTPIP51, silencing in HEK293 cells also reduced mitochondria–ER contacts by ∼50% (38). Therefore, it is possible that in both cases sufficient connections to the ER remain—allowing efficient lipid (VLCFA or PA) transfer in the presence of an appropriately positioned ACB/TPR domain.

However, our observations may also partially reflect the D3-C22:0 loading assay used for this work. In this assay exogenously added C22:0 would be expected to greatly exceed normal C22:0 levels. In this case, the ability of ACBD5 to recruit lipids to the peroxisomal membrane may be more critical than its ER-tethering function. Future assays, which more directly assess VLCFA transfer from the ER to peroxisomes, potentially utilizing novel trifunctional lipid probes, may give further insight into this process (39).

The ER-tethering capacity of ACBD5 may also be required for additional processes, including the transfer of membrane phospholipids for peroxisomal membrane expansion and the transport of ether (phospho)lipids (9, 20). In line with the former, we recently demonstrated that mutated ACBD5 proteins, which were unable to effectively interact with VAPB, showed reduced peroxisomal elongation upon ACBD5 expression in COS-7 cells (12). The synthesis of ether (phospho)lipids requires two intra-peroxisomal conversions followed by lipid transport from peroxisomes to ER. If this lipid transport would be dependent on the tethering function of ACBD5, one may expect a decrease in ether (phospho)lipids if ACBD5 is removed. Indeed, previous reports found decreased ether (phospho)lipids in ACBD5-deficient patient fibroblasts, HeLa cells with a knockdown in ACBD5 and cerebelli of Acbd5-deficient mice whilst no changes were detected in livers of Acbd5-deficient mice (14, 20). In the ACBD5 KO HEK293 cells, the total levels of ether (phospho) lipids did not change (Fig. 3*A*). For the Acbd5-deficient mice, the difference observed between cerebellum and liver were suggested to result from altered peroxisome proliferation in hepatocytes which might compensate for a less efficient synthesis pathway. We did not observe a clear proliferation in ACBD5 KO HEK293 cells, compared to wild-type HEK293 controls. Because ether-phospholipid production is expected to be high in brain, heart, spleen, and white blood cells, but relatively low in the liver (40), we speculate that it is possible that ether (phospho)lipid production in certain tissues or cells may be more dependent on ACBD5 activity than in others.

**Supporting Figure S3:**
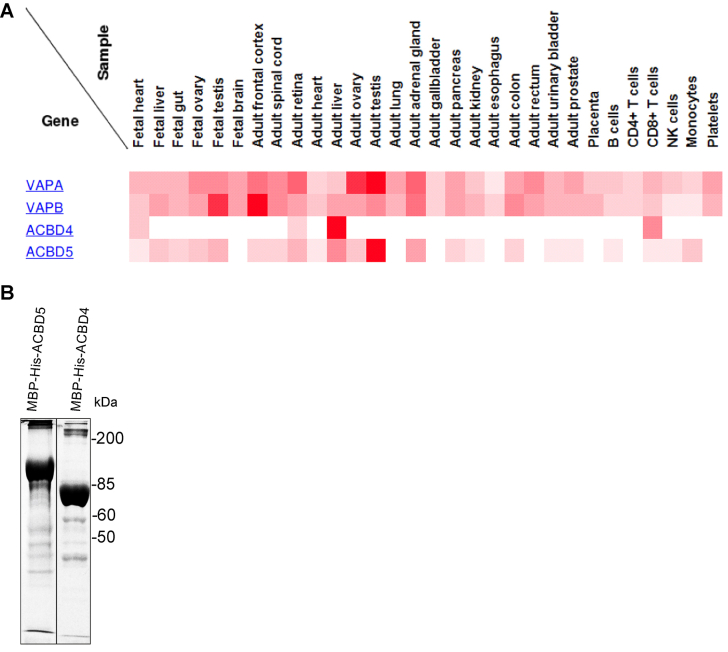
**ACBD4 and ACBD5 tissue expression pattern.***A*, expression data in different human tissues for VAPA, VAPB, ACBD4 and ACBD5 from the ISOEXPRESSO Database. *B*, coomassie blue stained SDS-PAGE gel of purified, recombinant MBP-His-ACBD5 and MBP-His-ACBD4 used for *in-vitro* binding experiments.

The observation that loss of ACBD4 did not cause a change in peroxisome–ER contacts but instead caused an apparent increase in VLCFA beta-oxidation is intriguing. One explanation for the lack of decreased peroxisome-ER tethering is that potentially lower expression levels of ACBD4 relative to ACBD5 mean that loss of ACBD4 does not significantly impact on the overall tethering forces. Indeed, a recent global mass spectrometry study in HEK293 cells estimated more than 30-fold higher protein levels of ACBD5 compared to ACBD4 (41). In terms of tissue expression, based on data in the IsoExpresso Database (Fig. S3) (42), ACBD5 appears to be broadly expressed in most tissues with potential enrichment in the testis and liver whereas ACBD4 may be more specifically expressed in the liver, heart, and retina. This is broadly in line with a recent analysis of human ACBD4 expression which suggested expression was highest in liver tissue (24) and a study using mouse tissues (20). Here, both ACBD4 and ACBD5 could be detected in all tissues tested but ACBD5 was enriched in the liver, while ACBD4 was only slightly enriched in the kidney and liver. However, in ACBD5-deficient mice, the loss of ACBD5 did not result in a compensatory increase in the levels of ACBD4 mRNA (20). Overall, this suggests that although both genes are expressed and show enrichment in liver tissue, overall levels of ACBD4 are significantly lower than ACBD5.

**Figure 7:**
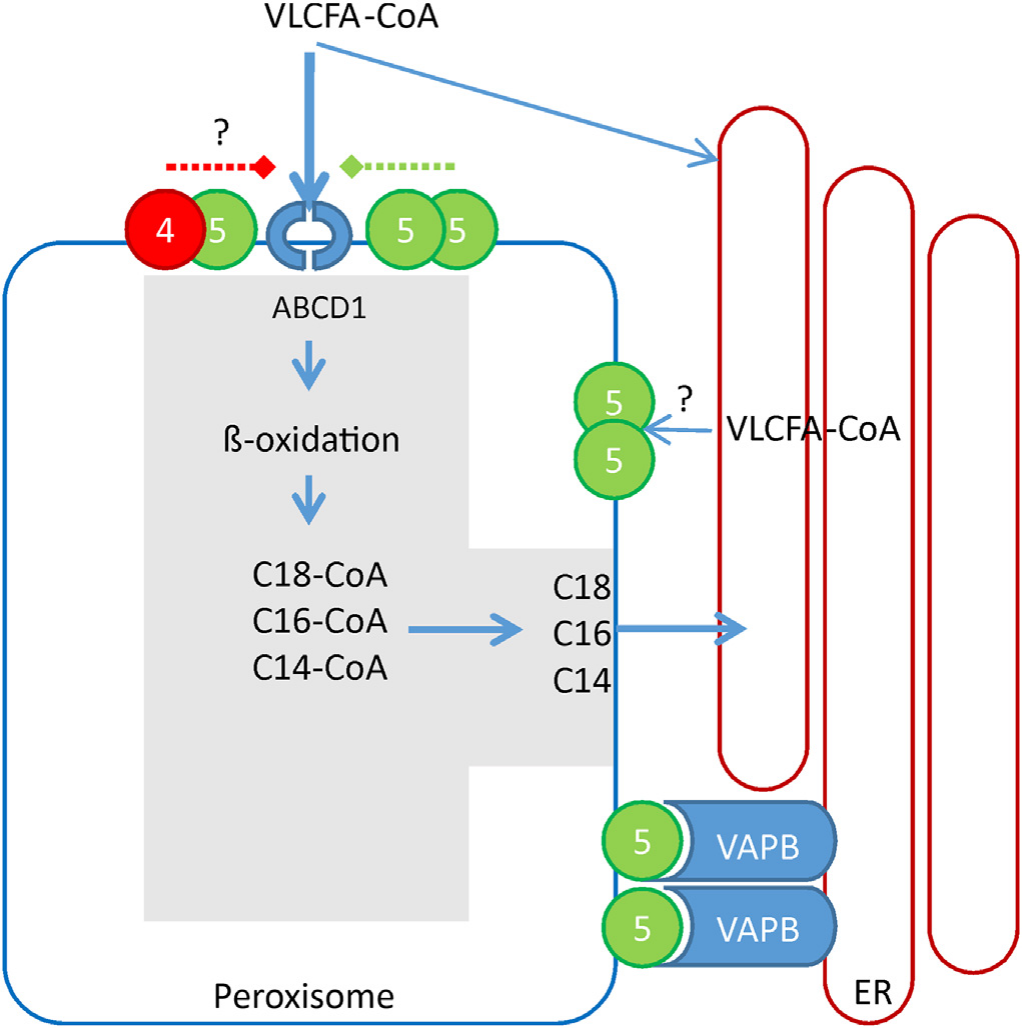
**Model for potential differential roles of ACBD4 and ACBD5.** At the peroxisome–ER interface, VLCFAs can be delivered to peroxisomes for beta-oxidation or retained on the ER membrane for elongation. Peroxisomal ACBD5 can act as a recruitment factor to deliver VLCFA-CoAs to the ABCD1 transporter for transfer to the peroxisomal lumen. Here, β-oxidation can generate medium chain-CoA substrates that can then be delivered back to the ER for desaturation. When ACBD5 is in complex with ACBD4, VLCFA-CoA recruitment may be inhibited. ACBD5 interaction with VAPB mediates tethering to the ER but the extent to which this interaction contributes to lipid flux between the organelles remains unclear.

Alternatively, ACBD4 may not play a significant role as a peroxisome-ER tether but instead has a more regulatory function. Overall, our data would be broadly consistent with a role for ACBD4 as a repressor of VLCFA processing in a manner that is dependent on the presence of ACBD5 (Fig. 7). In line with this, we observed an interaction between ACBD4 and ACBD5, which was independent of their ability to interact with VAPB. How an ACBD4/5 hybrid complex may functionally compare with the homodimeric ACBD4 and ACBD5 proteins is unclear, but one possibility would be altered VAPB interaction. However, we have shown that ACBD4 could complement VLCFA-processing defects in the absence of ACBD5 and that the tethering capacity (VAPB-binding) of ACBD5 was not required to restore VLCFA processing in ACBD5 KO cells. Therefore, it seems unlikely that any altered VAPB binding of an ACBD4–ACBD5 complex would result in the altered VLCFA processing we observed.

Another possibility, which might explain a repressive function of the ACBD4–ACBD5 heterodimer would be that its lipid-binding properties could be altered compared with the homodimeric proteins. In support of this, we observed a difference in substrate binding for the two homodimeric proteins, with ACBD5 preferentially interacting with the VLCFA C24-CoA relative to shorter chain substrates whilst ACBD4 appeared to show similar affinity for all the substrates tested (Fig. 5).

**Supporting Figure S4:**
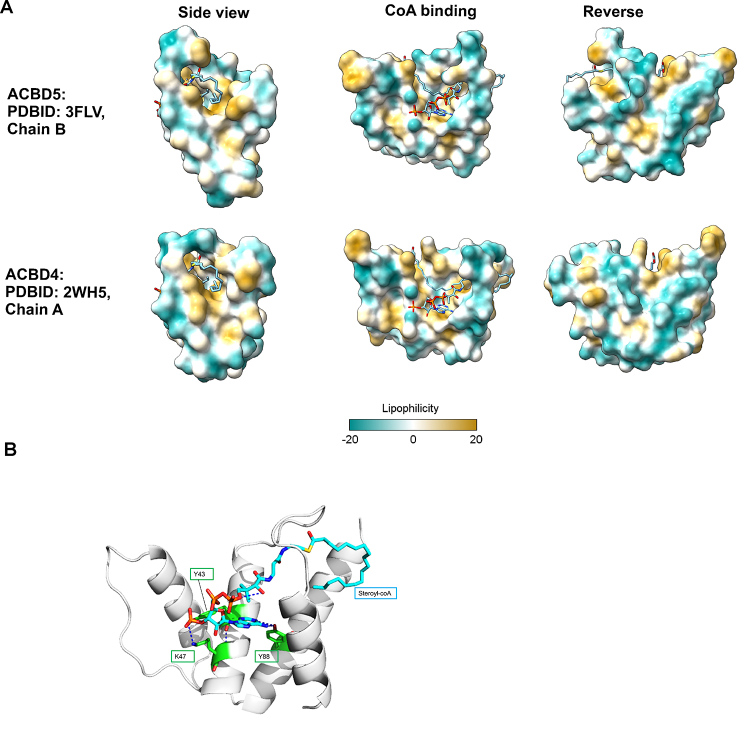
**Structural comparison of the ACB domain of ACBD4 and ACBD5.***A*, surface representation of the acyl-CoA binding domains of ACBD5 (PDBID: 3FLV:chainB) and ACBD4 (PDBID: 2WH5:chainA) coloured by lipophilicity. With cyan being the most lipophobic regions and gold the most lipophilic. Stearoyl-CoA is shown as bonds with grey (*C*) atoms. *B*, position of residues mutated in the ACB mutant of ACBD4. Figures generated using USCF ChimeraX.

To explore these differences in substrate binding preference further, we looked at the available unpublished structures of ACBD4 (PDBID: 2WH5) and ACBD5 (PDBID: 3FLV), in combination with Stearoyl-CoA, on the Protein Data Bank (https://www.ebi.ac.uk/pdbe/). Both ACBD4 and ACBD5 have largely similar CoA binding pockets with the fatty acid extending across the face of the protein (Fig. S4). There is a potential lipophilic patch in ACBD5 toward the reverse side of the protein, which is not present in ACBD4 and may correlate with the expected position of a longer chain substrate. However, these structures have been determined using Stearoyl-CoA, so without extensive modeling or further structural studies, it is unclear if this is a significant difference, but this does provide a possible direction for future studies. An alternative hypothesis is that dimerization of ACBD5 allows the fatty acid chain to bind across both monomers, as is seen in the structure for ACBD1 (PDBID: 2CB8). Here, myristoyl-CoA is shown to bind across two ACBD1 monomers. If this were the case and an ACBD4/5 heterodimer had an altered dimerization interface, then this might result in altered substrate binding compared with the individual proteins and could potentially explain the possible repressive role observed for ACBD4.

Overall, the mechanism by which ACBD4 might repress VLCFA processing remains unclear. However, as increased expression of ACBD4 can compensate for loss of ACBD5, this opens up the possibility of a potential therapeutic approach for ACBD5 deficiency. This can be envisaged in a similar way to the concept of increasing expression of the alternative VLCFA transporter ABCD2 as a therapeutic approach for the loss of ABCD1 in X-ALD (43).

As the cellular lipid substrates of ACBD4 and ACBD5 are still unclear and a formal demonstration of their mechanism of action at membrane contact sites is also lacking, future work should focus on investigating this along with defining a clear cellular role for ACBD4 and investigating possible mechanisms to upregulate ACBD4 expression as a potential therapeutic approach in ACBD5 deficiency.

## Experimental procedures

### Plasmids and antibodies

Site-directed mutagenesis was performed with the QuikChange XL Kit (Agilent) according to the manufacturer’s instructions. To construct pcDNA5/frt vectors expressing ABCD4 and ABCD5 for use in the FlpIn system, the coding sequences for ACBD5 and mutants were released as EcoRV-XhoI fragments and the coding sequences for ACBD4 and mutants were released as HindIII-BamHI fragments from pCMV-Tag2B versions and cloned into corresponding sites of pcDNA5/frt. See Tables S1–S4 for details of the plasmids used in this study.

See Table S5 for details of antibodies used in this study.

Note. Both ACBD4 and ACBD5 have several potential different isoforms, including some isoforms that do not contain predicted C-terminal TMDs or FFAT motifs. For simplicity in this study, we used the isoforms that have been previously published (4, 9) and refer to Uniprot nomenclature with ACBD4iso2 (Q8NC06–2) and ACBD5iso2 (**Q5T8D3-2**), with amino acid numbers referring to those used in these isoforms.

### Cell culture and transfection

COS-7 (African green monkey kidney cells; ATCC CRL-1651) cells were cultured in Dulbecco’s modified Eagle’s medium (DMEM), with high glucose (4.5 g/l) supplemented with 10% fetal bovine serum (FBS), penicillin and streptomycin at 37 °C with 5% CO_2_ and 95% humidity and transfected using diethylaminoethyl (DEAE)-dextran (Sigma-Aldrich). HEK293 (human embryonic kidney derived; ATCC CRL-1573) cells were cultured at 37 °C with 5% CO_2_ in DMEM supplemented with L-glutamine (BioWhittaker), 10% fetal bovine serum (ThermoFisher), 25 mM HEPES buffer (BioWhittaker), 100 U/ml penicillin (ThermoFisher), 100 μg/ml streptomycin (ThermoFisher) and 250 ng/ml Fungizone (ThermoFisher).

### Generation of HEK293 KO and FlpIN cell lines

ACBD4 and ACBD5 KOs were made by CRISPR-Cas9 genome editing technology according to the protocol of Ran *et al.* (44). The guides used to generate ACBD4 KOs are 5′-AGTCCAGGTCCCTGGGTGAA-′3 and 5′-GTGAATGGGACTCTGTGGAG-′3 (targeting exon 9) and for ACBD5 Kos 5′-ACGTGCTCTGATCCAAACTC-′3 (targeting exon 2). The guides were cloned into the pSpCas9(BB)-2A-GFP vector (Addgene plasmid ID: 48138). Cultured HEK293 cells were transfected with jetPRIME (Polyplus transfection) after which GFP-expressing cells were FACS sorted and plated one cell per well in a 96 wells plate and incubated at 37 °C in DMEM medium as described above. After 6 to 8 weeks the respective gene KOs were confirmed by Sanger sequence analysis. To this end, genomic DNA was isolated from the cells using Phire Animal Tissue Direct PCR Kit (ThermoFisher). After PCR amplification of exon 9 of ACBD4 and exon 2 of ACBD5 using gene-specific primers tagged with a −21M13 (5′ TGTAAAACGACGGCCAGT-3′) or an M13rev (5′-CAGGAAACAGCTATGACC-3′) sequence, the PCR products where sequenced with −21M13 or M13rev primers. Sequence analysis was performed using the Big DyeTM Terminator v.3.1 Cycle Sequencing Kit on an ABI 3730 sequencer (Applied Biosystems).

Wild-type ACBD4 and ACBD5 and different mutants were stably overexpressed following genomic integration of their coding cDNA sequences into a FlpIn site. The FlpIn site was introduced using the FlpIn system of ThermoFisher according to the manufacturer’s protocol. The cell lines containing the FlpIn site are labeled throughout with the number 2. The coding cDNAs of wild-type ACBD4 and ACBD5 and different mutants were cloned into the pcDNA5/frt vector and transfected together with pOG44 in HEK293 FlpIn cells using jetPRIME. Cells were plated in 96 well plates and selected on 150 μg/ml hygromycin (Invitrogen) until stable expression clones were obtained. Overexpression was checked by immunoblotting (Fig. S2).

### Immunoprecipitation and immunoblotting

For protein interaction studies, Myc tagged and/or FLAG-tagged proteins were expressed in COS-7 cells for 48 h. Cells were chilled on ice and then lysed in lysis buffer (50 mM Tris-HCL pH 7.4, 150 mM NaCl, 1% Triton X-100, and protease inhibitor cocktail) followed by centrifugation at 15,000*g*. Lysates were incubated with Myc-TRAP (ChromoTek) for 1 h at 4 °C. The affinity beads were then washed with lysis buffer and bound proteins were eluted with Laemmli buffer. Following separation by standard SDS-PAGE, proteins (IP samples and total lysate inputs) were analyzed by immunoblotting using antibodies as indicated in Table S5. A signal was detected *via* enhanced chemiluminescence reagents (Amersham Bioscience) using Amersham hyperfilm (GE Healthcare) or the G:Box Chemi (Syngene).

### *E.coli* expression, purification, and *in vitro* binding assay

For *in vitro* binding assays (Fig. S1), GST-VAPBmsp domain and His-MBP-ACBD4 constructs (lacking the TMD) were expressed in BL21 Rosetta (DE3) cells (EMD Millipore). Expression was induced with 0.1 mM IPTG at 18 °C for 24 h. Cells were harvested by centrifugation at 5000*g* for 10 min at 4 °C. Cell pellets were resuspended in ice-cold lysis buffer (50 mM Tris-HCl, pH7.4, 150 mM NaCl, 1 mM DTT, 0.5 mM EDTA and cOmplete, Mini Protease Inhibitor Cocktail (Roche) and disrupted by sonication. Insoluble material was removed by centrifugation at 20,000*g* for 15 min.

VAPB was purified on a 1 ml HiTrap Glutathione Sepharose 4B column (Cytiva) equilibrated in 50 mM Tris-HCl, pH 7.4, 150 mM NaCl, 1 mM DTT, 0.5 mM EDTA connected to an AKTA PrimePlus (Cytiva). 40 units PreScission Protease (Cytiva) diluted to 1 ml in 50 mM Tris-HCl pH 7.4, 150 mM NaCl was added to the column and incubated overnight at 4 °C. Cleaved purified protein was eluted with 50 mM Tris-HCl, pH 7.4, 150 mM NaCl.

ACBD4 was purified on a 1 ml HiTrap MBPtag column (Cytiva) equilibrated in 50 mM Tris-HCl pH 7.4, 300 mM NaCl, 10 mM Imidazole connected to an AKTA PrimePlus. ACBD4 was eluted with 10 mM Maltose in 50 mM Tris-HCl, pH 7.4, 300 mM NaCl, 10 mM Imidazole.

Purified ACBD4 at 10 μM was incubated with HisPur Ni-NTA Resin (ThermoFisher) for 1 h on a rotating shaker at 4 °C. The protein-bound resin was incubated with purified VAPB for 1 h. Beads were washed extensively with wash buffer (50 mM Tris-HCl, pH 7.4, 300 mM NaCl, 10 mM Imidazole) and proteins were then eluted with 0.5 M imidazole in 50 mM Tris-HCl, pH 7.4, 300 mM NaCl and analyzed by immunoblotting.

### Electron microscopy and spatial stereology

Electron microscopy was performed as described previously (9). Monolayers of cells were fixed in 0.5% glutaraldehyde in 0.2 M PIPES buffer (pH 7.2), and post-fixed in 1% osmium tetroxide (reduced with 1.5% w/v potassium ferrocyanide) in cacodylate buffer. Following washing in deionized water, cells were dehydrated in a graded ethanol series before embedding in Durcupan resin (Sigma Aldrich). 60 nm ultra-thin sections were collected on pioloform-coated 100 mesh copper EM grids (Agar Scientific) and contrasted with lead citrate. Imaging was performed with a JEOL JEM 1400 transmission electron microscope and images were acquired with an ES 1000W CCD, Gatan digital camera.

Quantification of peroxisome-ER contacts was also performed as previously (9). Peroxisomes were identified by size, the presence of a single membrane, and a homogenous fine-granular matrix. This was based on the morphology and size of peroxisomes labeled with GFP-PTS1 by immunogold EM (45). Peroxisomes were sampled (mean = 56 ± 2 (S.E.M.) peroxisomes per grid) by scanning EM grids systematic uniform random. To estimate the mean fraction of the total peroxisome membrane surface in direct contact with the ER, a stereological approach by line intersection counting was used. Intersections were classified as direct membrane contact (defined as “attachment”) if there was <15 nm distance between peroxisome and ER membranes.

### Metabolic and biochemical analyses

The concentrations of VLCFAs were measured as previously described (46, 47) and C16:0 β-oxidation was determined as described in (48). For the C16:0 β-oxidation measurements, 10 μM 2-[5-(4-chlorophenyl)pentyl]oxirane-2-carboxylate (POCA) was added to inhibit the mitochondrial β-oxidation. A D3-C22:0 loading test was performed, essentially as previously described (32), by loading cells for 3 days with deuterated (D3) C22:0 followed by fatty acid analysis with tandem mass spectrometry.

### Lipidomics

For lipidomics, each cell line was cultured in triplicate in DMEM supplemented with L-glutamine, 10% fetal bovine serum, 25 mM HEPES buffer, 100 U/ml penicillin, 100 μg/ml streptomycin and 250 ng/ml Fungizone at 37 °C under an atmosphere of 5% CO_2_. After they reached confluence, the cells were harvested and prepared for lipidomics. To this end, lipids were extracted, and analyzed and data were processed by the core Facility Metablomics (Amsterdam UMC) as described by Vaz *et al.* (49).

### Acyl-CoA binding experiments

*In vitro* binding assays were performed with purified ACBD4 and ACBD5 protein (2 μM) as previously described (34). Briefly, MBP-His tagged ACBD4 and ACBD5 were purified with amylose resin (See Fig. S3) and competition experiments were then performed with increasing concentration of C16:0-CoA, C18:1-CoA or C24:0-CoA (0–20 μM) mixed with [^14^C]C18:1-CoA (5 μM) prior to the addition of the protein. Reactions were then pulled down with NTA 50% slurry at 4 °C for 10 min. After washing the amount of [14C]C18:1-CoA in the bound-resin fraction was quantified with a scintillation counter.

### Statistical analyses

The stereological data were statistically tested by one-way analysis of variance with Tukey’s multiple comparison test. VLCFA data were analyzed with two-tailed unpaired *t* test (according to the legends of each figure). GraphPad Prism was used for analysis.

## Data availability

The research data supporting this publication are provided within this paper, or as Supplementary information.

## Supporting information

This article contains supporting information (4, 9, 12, 23).

## Conflict of interest

All authors declare that they have no conflict of interest.
