# Supplementary material for: Differential roles for ACBD4 and ACBD5 in peroxisome–ER interactions and lipid metabolism
Source: J Biol Chem. 2023 Jul 4;299(8):105013. doi: 10.1016/j.jbc.2023.105013 (PMC10410513; doi:10.1016/j.jbc.2023.105013)
Supplement: Supporting Table S1 [file mmc1.docx]

| Plasmid | Source | Vector |
| --- | --- | --- |
| FLAG-ACBD4 | (23) | pCMV-Tag2B |
| FLAG-ACBD5 | (9) | pCMV-Tag2B |
| Myc-ACBD4 | (4) | pCMV-Tag3B |
| Myc-ACBD5 | (9) | pCMV-Tag3B |
| Myc-VAPB | C. Miller, King’s College London, London, UK |  |
| GST-VAPB  FLAG-ACBD4 FFAT | (9)  (12) | pGEX-6p2  pCMV-Tag2B |
| FLAG-ACBD5 FFAT | (12) | pCMV-Tag2B |
| MBP-His-ACBD5 | (9) | pETM41 |

**Table S1. Plasmids used in this study**
