# Supplementary material for: Differential roles for ACBD4 and ACBD5 in peroxisome–ER interactions and lipid metabolism
Source: J Biol Chem. 2023 Jul 4;299(8):105013. doi: 10.1016/j.jbc.2023.105013 (PMC10410513; doi:10.1016/j.jbc.2023.105013)
Supplement: Supporting Table S2 [file mmc2.docx]

**Table S2. Plasmids generated in this study**

| Plasmid | Template | Primers | Enzymes | Vector |
| --- | --- | --- | --- | --- |
| FLAG-ACBD5 CC  (M416P) | FLAG-ACBD5 | A5_CC_1_Fw  A5_CC_1_Rv |  | pCMV-Tag2B |
| FLAG-ACBD4 AcB  (Y43F, K47A, Y88A) | FLAG-ACBD4 | A4_ACB_1_Fw  A4_ACB_1_Rv  A4_ACB_2_Fw  A4_ACB_2_Rv  A4_ACB_3_Fw  A4_ACB_3_Rv |  | pCMV-Tag2B |
| FLAG-ACBD4 CC  (M244P) | FLAG-ACBD4 | A4_CC_1_Fw  A4_CC_1_Rv |  | pCMV-Tag2B |
| MBP-His-ACBD4 | Gene synthesis (Eurofins) |  | NcoI, BamHI | pETM41 |
| GST-VAPBmsp | GST-VAPB | VAPBmsp_Fw  VAPBmsp_Rv |  | pGEX-6p2 |

Numbering is according to the nucleotide sequence of ACBD4 isoform 2 (UniProt identifier: Q8NC06-2) and ACBD5 isoform 2 (Q5T8D3-2).
