# Supplementary material for: Differential roles for ACBD4 and ACBD5 in peroxisome–ER interactions and lipid metabolism
Source: J Biol Chem. 2023 Jul 4;299(8):105013. doi: 10.1016/j.jbc.2023.105013 (PMC10410513; doi:10.1016/j.jbc.2023.105013)
Supplement: Supporting Table S3 [file mmc3.docx]

**Table S3. Primers used in this study**

| Primer | Sequence |
| --- | --- |
| A4_CC_1_Fw  A4_CC_1_Rv | GCACTACAGGAGAGCCCGCAGGAGGTGCAGGCG  CGCCTGCACCTCCTGCGGGCTCTCCTGTAGTGC |
| A5_CC_1_Fw  A5_CC_1_Rv | AGACTGCAGGAGGACCCGCAGAATGTCCTTCAG  CTGAAGGACATTCTGCGGGTCCTCCTGCAGTCT |
| A4_ACB_1_Fw  A4_ACB_1_Rv | GAGATGCTGCGATTCTTCAGTTACTACAAGCAGGC  GCCTGCTTGTAGTAACTGAAGAATCGCAGCATCTC |
| A4_ACB_2_Fw  A4_ACB_2_Rv | CGATTCTTCAGTTACTACGCGCAGGCCACCATGGG  CCCATGGTGGCCTGCGCGTAGTAACTGAAGAATCG |
| A4_ACB_3_Fw  A4_ACB_3_Rv | GAGGCCATGTCTGCCGCCATCACTGAAATGAAAC  GTTTCATTTCAGTGATGGCGGCAGACATGGCCTC |
| VAPBmsp_Fw  VAPBmsp_Rv | TAGATGTGTGTTTGAATAGCCAGCAGAGAATG  CATTCTCTGCTGGCTATTCAAACACACATCTA |
