# Supplementary material for: Differential roles for ACBD4 and ACBD5 in peroxisome–ER interactions and lipid metabolism
Source: J Biol Chem. 2023 Jul 4;299(8):105013. doi: 10.1016/j.jbc.2023.105013 (PMC10410513; doi:10.1016/j.jbc.2023.105013)
Supplement: Supporting Table S4 [file mmc4.docx]

**Table S4. Codon optimized ACBD4 for expression in E. coli**

| Gene | Source | Sequence (5′ to 3′) |
| --- | --- | --- |
| ACBD4-codon-optimized | Eurofins Genomics | CCATGGAC ATG GGT ACG GAG AAA GAA TCT CCG GAG CCG GAT TGC CAG AAA CAG TTT CAA GCC GCT GTT TCC GTG ATC CAG AAT CTG CCC AAG AAC GGG AGC TAT CGC CCG TCA TAT GAA GAA ATG CTG CGC TTT TAC TCC TAC TAC AAA CAA GCG ACT ATG GGA CCT TGC TTA GTT CCG CGT CCA GGC TTT TGG GAC CCG ATT GGC CGG TAT AAG TGG GAT GCC TGG AAC TCG TTA GGC AAA ATG AGC CGT GAA GAG GCG ATG AGT GCG TAC ATT ACC GAG ATG AAA CTG GTT GCG CAG AAA GTG ATC GAT ACC GTC CCT CTT GGA GAA GTT GCG GAA GAT ATG TTC GGC TAT TTC GAA CCG CTG TAT CAG GTC ATT CCC GAC ATG CCA CGT CCG CCC GAA ACG TTT CTC CGT CGC GTG ACA GGT TGG AAA GAG CAA GTC GTG AAT GGG GAT GTG GGT GCA GTG AGC GAA CCT CCA TGT CTG CCG AAA GAG CCT GCT CCT CCG TCA CCG GAA AGC CAT TCA CCG CGC GAT CTT GAC TCC GAG GTG TTC TGT GAT AGC CTG GAA CAG CTG GAA CCG GAA CTG GTA TGG ACC GAA CAG CGC GCA GCA TCT GGC GGC AAA CGC GAT CCG CGC AAC AGT CCT GTT CCG CCG ACC AAG AAA GAA GGT CTG CGC GGT TCG CCA CCC GGT CCC CAG GAA TTG GAC GTA TGG CTG TTG GGC ACT GTA CGT GCC TTA CAG GAG TCG ATG CAA GAA GTG CAG GCT CGT GTC CAG AGC CTC GAA AGT ATG CCG CGT CCA CCG GAA CAA CGC CCA CAA CCG CGC CCA TCT GCC CGG CCG TAA CCC CTT GGG CTC CCG GGG CCC GCG CTG CTC TTC TTC CTC CTG TGG CCC TTC GTC GTC CAG TGG CTC TTC CGA ATG TTT CGG ACC CAA AAG AGG TGA GGATCC |
