# Supplementary material for: Differential roles for ACBD4 and ACBD5 in peroxisome–ER interactions and lipid metabolism
Source: J Biol Chem. 2023 Jul 4;299(8):105013. doi: 10.1016/j.jbc.2023.105013 (PMC10410513; doi:10.1016/j.jbc.2023.105013)
Supplement: Supporting Table S5 [file mmc5.docx]

**Table S5. Primary and secondary antibodies used in this study**

| Antibody | Type | Dilution | Source |
| --- | --- | --- | --- |
| FLAG | mc ms | 1:2000 | Sigma F3165 |
| FLAG | pc rb | 1:1000 | Sigma F7425 |
| Myc | mc rb | 1:2000 | Abcam ab9106 |
| polyHistidine−Peroxidase (conjugated) | mc ms | 1:2000 | Sigma A7058 |
| ACBD4 | pc rb | 1:1000 | Proteintech 20941-1-AP |
| ACBD5 | pc rb | 1:1000 | Cambridge Bioscience HPA012145 |
| VAPB | pc rb | 1:500 | Abcam ab103638 |
| VAPB | pc rb | 1:1,000 | Sigma HPA013144 |
| β-Actin | mc ms | 1:10000 | Sigma A5441 |
| HRP IgG | gt anti-rb | 1:10,000 | Bio-Rad Laboratories 170-6515 |
| HRP IgG | gt anti-ms | 1:10,000 | Bio-Rad Laboratories 170-6516 |

Abbreviations: mc, monoclonal; pc, polyclonal; ms, mouse; rb, rabbit; gt, goat; HRP, horseradish peroxidase.
